# Orbital and Ophthalmologic Injuries Associated With Standing Electric Scooter Accidents: A Narrative Synthesis of Clinical Outcomes and Injury Patterns

**DOI:** 10.7759/cureus.102447

**Published:** 2026-01-27

**Authors:** Mohammed Haque, Lily Hoque

**Affiliations:** 1 Accident and Emergency, Calderdale and Huddersfield NHS Foundation Trust, Bradford, GBR; 2 General Practice, Bradford Teaching Hospitals NHS Foundation Trust, Bradford, GBR

**Keywords:** craniofacial injuries, electric scooter, emergency orbital decompression, e-scooter accidents, facial trauma, micromobility injuries, oculoplastic surgery, orbital fracture, retrobulbar hemorrhage, vision loss

## Abstract

Standing electric scooters (e-scooters) have introduced a distinct pattern of facial trauma to emergency departments worldwide. Their unique biomechanics, with riders standing upright on a narrow platform, creates a characteristic forward pitching mechanism during sudden stops that directs high-energy impacts to the anterior midface and orbital region. This differs fundamentally from bicycle accidents, where lateral falls and arm bracing are typical. This review synthesises available clinical evidence on orbital and ophthalmologic injuries from electric scooter accidents. Twelve primary studies were identified, encompassing 1,675 patients across diverse geographic settings in the clinical cohorts. Orbital fractures commonly involve multiple walls simultaneously, with the floor and lateral wall most frequently affected. Vision-threatening complications, although infrequent, are clinically significant. Orbital compartment syndrome represents a time-critical emergency requiring immediate lateral canthotomy and cantholysis to prevent irreversible vision loss. Retrobulbar haemorrhage and retinal injuries also occur, although globe rupture is rare. Soft tissue injuries, including facial lacerations and eyelid trauma, are more common than fractures. Alcohol intoxication and low helmet use emerge as key modifiable risk factors associated with increased injury severity. These findings emphasise the need for high clinical suspicion for orbital pathology in electric scooter trauma and highlight opportunities for targeted prevention strategies.

## Introduction and background

The rapid proliferation of dockless electric scooters (e-scooters) since their widespread deployment in 2017 has fundamentally transformed urban micromobility [1-3]. Initially launched by companies such as Bird and Lime in select United States cities, e-scooter sharing systems have expanded globally and are now ubiquitous in metropolitan centres across North America, Europe and Asia-Pacific. Market projections indicate continued exponential growth over the next decade [2]. Whilst these devices offer convenient last-mile transportation solutions, their integration into the urban environment has introduced a novel and concerning pattern of traumatic injuries.

Amongst the spectrum of e-scooter-related trauma, craniofacial injuries have emerged as particularly prevalent and severe [4-7]. Within this category, orbital and ophthalmologic trauma represents a subset, accounting for approximately 25% of e-scooter facial fractures [6], and requires further characterisation due to the potential for permanent vision loss and functional disability.

Biomechanics and injury mechanisms

The unique biomechanics of e-scooter accidents fundamentally differ from traditional micromobility injuries. E-scooter riders stand on a platform with a high centre of gravity. The e-scooter's standing configuration and lack of handlebar bracing options predispose riders to unprotected anterior facial impact, directly exposing the midface and orbital region to concentrated blunt force [6,7]. Evidence from parallel micromobility studies underscores the impact of motorised velocity on trauma severity; e-bike riders sustain midface fractures more frequently than conventional cyclists (47% versus 34%) [8] and demonstrate higher fracture complexity ratios (4.25 versus 2.34 fracture lines per case) [9]. When deceleration occurs, the forward momentum causes a characteristic "pitch-over" mechanism, in which the rider's centre of mass moves forward faster than their feet can travel, resulting in direct high-energy anterior facial impact against the ground or fixed objects [10,11]. This contrasts sharply with bicycle injuries, where riders either fall laterally to the side (resulting in lateral facial impacts) or extend their arms instinctively to brace falls [11]. Bicycle falls result in upper extremity trauma and lateral facial trauma as secondary consequences of arm positioning.

Current evidence and knowledge gaps

Emergency departments worldwide have reported a disproportionate rise in orbital fractures and associated ocular complications amongst e-scooter users compared to other micromobility cohorts [12,13]. Orbital fractures occur in approximately 21% of e-scooter-related ophthalmic injuries versus only 4% in conventional (non-powered) scooter riders, a five-fold increased risk [14]. When compared to conventional bicycles, e-scooter riders exhibit fundamental biomechanical and demographic differences. This represents a five-fold difference reflecting the distinct injury biomechanics.

However, detailed characterisation of orbital trauma prevalence, specific fracture patterns, vision-threatening complications and ophthalmologic management outcomes specific to e-scooter accidents remains limited. Most existing reviews focus on general craniofacial injury patterns without providing granular orbital-specific data, surgical outcomes or functional visual results. Consequently, current management often defaults to broad trauma algorithms, such as Advanced Trauma Life Support (ATLS) [15], which prioritise life-threatening pathology but may not adequately address the distinct biomechanics and vision-threatening risks of e-scooter trauma. This creates a critical evidence gap for ophthalmologists and emergency medicine physicians who increasingly encounter these patients in acute care settings but lack evidence-based guidance on orbital injury assessment, imaging decision-making, surgical indications and prognostic counselling specific to the e-scooter injury pattern.

Objectives and scope

This narrative review synthesises findings from peer-reviewed international studies to comprehensively characterise orbital and ophthalmologic injuries associated with e-scooter accidents. Specific objectives are to (1) define the prevalence and anatomical distribution of orbital fractures in e-scooter trauma, (2) describe the spectrum of vision-threatening complications and their emergency management, (3) quantify the surgical burden of orbital trauma and review management approaches and (4) identify key modifiable and non-modifiable risk factors associated with severe orbital injury. By establishing a clear clinical profile of e-scooter-related orbital trauma, this review aims to inform ophthalmologic practice patterns, guide clinical decision-making in the acute setting, highlight critical evidence gaps requiring future investigation and inform public health prevention strategies addressing this emerging injury pattern.

## Review

Methods

Study Design and Protocol

This is a narrative synthesis of peer-reviewed clinical studies examining orbital and ophthalmologic injuries associated with accidents involving standing electric scooters. The review was conducted using standard principles for evidence synthesis. The protocol was not prospectively registered prior to initiation; however, the methodology, inclusion/exclusion criteria and evidence synthesis approach were specified in advance within an internal protocol draft established prior to data extraction. This represents a narrative rather than a systematic review, given the presence of some heterogeneity in study designs, outcome measures and patient populations.

Search Strategy

A comprehensive literature search was conducted across PubMed/MEDLINE and Google Scholar databases to identify all published studies reporting orbital or ophthalmologic trauma associated with standing electric scooter use. The search strategy employed Medical Subject Headings (MeSH) terms combined with free-text keywords to maximise sensitivity whilst maintaining specificity for orbital injury outcomes.

The PubMed search string was as follows: ("electric scooter"[Title/Abstract] OR "e-scooter"[Title/Abstract] OR "e scooter"[Title/Abstract] OR "standing scooter"[Title/Abstract] OR "shared scooter"[Title/Abstract]) AND ("injury"[Title/Abstract] OR "trauma"[Title/Abstract] OR "fracture"[Title/Abstract] OR "craniofacial"[Title/Abstract] OR "maxillofacial"[Title/Abstract] OR "facial"[Title/Abstract] OR "orbital"[Title/Abstract] OR "ocular"[Title/Abstract] OR "ophthalmologic"[Title/Abstract] OR "vision loss"[Title/Abstract] OR "head injury"[Title/Abstract]).

A parallel search was conducted in Google Scholar using comparable terminology. Given the orbital focus, supplementary searches incorporated specific terms including "orbital fracture", "ocular injury", "ophthalmologic trauma", "retrobulbar haemorrhage", "compartment syndrome" and "vision loss." All searches were restricted to peer-reviewed articles published in English between January 2017 and December 2024, reflecting the period of rapid global expansion of dockless e-scooter sharing systems. The search was intentionally limited to PubMed and Google Scholar; we did not search Embase, the Cochrane Library or other specialised databases, representing a potential limitation in comprehensiveness of source capture.

Study Selection and Screening Process

**Figure 1:**
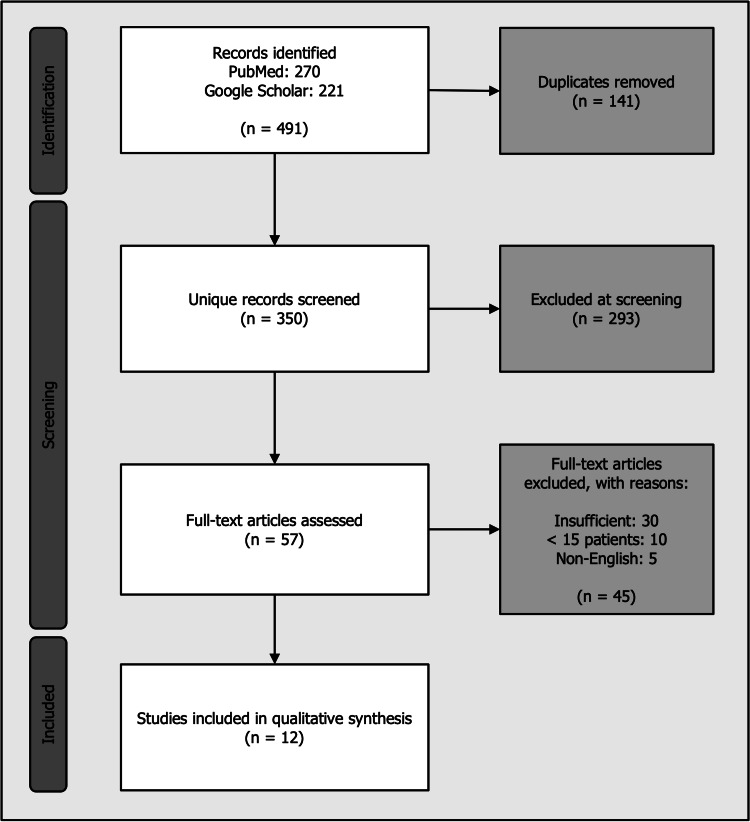
Study Selection Process and Search Results

The initial search identified 270 records from PubMed and 221 from Google Scholar (N=491). After removal of duplicates (n=141), 350 unique records underwent independent title and abstract screening by both authors. The authors assessed each abstract independently against predefined inclusion and exclusion criteria. Disagreements regarding inclusion at the abstract screening stage were resolved through discussion and consensus. Following initial screening, 57 full-text articles were retrieved and independently assessed for eligibility by both reviewers. Any disagreements at full-text review were resolved through detailed discussion and consensus, with no requirement for third-party adjudication. Following application of predetermined inclusion and exclusion criteria, 12 primary studies meeting all inclusion criteria were selected for detailed narrative synthesis, published between 2019 and 2024, with data collection periods spanning 2017-2023 (Figure 1).

Inclusion Criteria

Studies were included if they reported orbital, ocular or ophthalmologic injuries specifically resulting from standing electric scooter accidents. Studies had to provide extractable, granular data on orbital fracture patterns and anatomical location. Alternatively, eligible studies could contribute data on ocular complications such as retrobulbar haemorrhage, globe integrity, visual acuity and intraretinal injury, or data on surgical management and outcomes of orbital trauma. Eligible study designs comprised original research articles including case series, cohort studies, cross-sectional studies and prospective observational studies. All studies enrolled a minimum of 15 patients with documented craniofacial injuries and were published as full-text, peer-reviewed articles in the English language. Studies were classified as primary research if they involved original data collection and analysis at the patient level. This included cohort studies, case series and cross-sectional studies.

Exclusion Criteria

Studies were excluded if they focused exclusively on orthopaedic or extremity trauma without extractable craniofacial or orbital-specific data. Additionally, studies were excluded if they exclusively examined non-standing scooters, non-motorised scooters, mopeds, motorcycles or seated mobility devices only. Secondary research articles, including systematic reviews, scoping reviews, narrative reviews and meta-analyses, were excluded, as our objective was to synthesise primary research data rather than re-analyse previously published syntheses. Studies were also excluded if they were published as conference abstracts, editorials, opinion pieces or letters without full-text peer-reviewed publication, or if they were published in languages other than English. Finally, studies providing insufficient granularity in orbital or ocular injury classification or representing duplicate publications of previously identified studies were excluded.

Data Extraction

Data extraction was performed independently by both authors using a standardised protocol specifically focused on orbital and ophthalmologic outcomes. Each author extracted data from a subset of included studies, with systematic cross-verification of approximately 30% of extractions to ensure consistency and accuracy. Any discrepancies identified during verification were resolved through discussion and re-review of the source document. Variables systematically extracted included study characteristics (country, design, setting and study period), orbital fracture anatomical location by orbital wall (floor, medial, lateral and roof) and fracture prevalence using both patient-based and fracture-based denominators. Specific attention was paid to distinguishing isolated orbital wall fractures from component fractures of the zygomaticomaxillary complex (ZMC) or naso-orbital-ethmoid (NOE) complex; Le Fort fractures, particularly types 2 and 3, were documented as these frequently involve orbital structures in conjunction with NOE injuries and midface trauma. Vision-threatening complications extracted included orbital compartment syndrome, retrobulbar haemorrhage, globe rupture, traumatic optic neuropathy and retinal injuries, with visual acuity documented at presentation and follow-up when available. Surgical data included intervention rates, specific procedures, timing, operative approach, implant materials and postoperative complications. Soft tissue injuries encompassed eyelid lacerations, periorbital contusions and repair requirements. Risk factors included patient age, sex, alcohol intoxication status, helmet use, temporal injury patterns, injury mechanism and environmental factors. Study quality characteristics, including follow-up duration and data completeness, were documented.

Evidence Synthesis Approach

Given the observational nature of all included studies and substantial heterogeneity in study design, outcome measures, patient populations, surgical indication thresholds and geographic/temporal context, a structured narrative synthesis approach was adopted to (1) construct a unified clinical profile of e-scooter-related orbital and ophthalmologic trauma by identifying consistent injury patterns across diverse clinical settings, (2) characterise the range of prevalence estimates for orbital fractures and associated complications with explicit discussion of why estimates vary across studies, (3) identify and describe vision-threatening complications requiring emergency intervention and (4) characterise key modifiable and non-modifiable risk factors associated with severe orbital injury. Prevalence estimates are presented with ranges and explicit notes regarding study-specific denominators and cohort composition to allow readers to interpret findings in the appropriate context. Evidence gaps are explicitly identified throughout the synthesis, distinguishing between findings replicated across multiple independent studies, findings reported by single studies and areas where no data are available in the literature.

Statistical Analysis

Pooled prevalence estimates were calculated using simple proportion methods, summing numerators and denominators across studies with comparable populations and outcome definitions. Ninety-five percent confidence intervals for pooled proportions were calculated using the Wilson score method. Heterogeneity across studies was assessed by examining ranges in reported prevalence and by qualitative evaluation of study population differences, denominator definitions and methodological variations. Substantial methodological heterogeneity precluded formal meta-analysis using random-effects or fixed-effects models; therefore, we present pooled estimates alongside ranges and explicit discussion of sources of heterogeneity to allow appropriate interpretation. When studies reported outcomes using different denominators (e.g., proportion of all injuries versus proportion of craniofacial injuries), these were analysed separately and clearly distinguished in presentation of results. Odds ratios and statistical associations reported by individual studies are presented as originally calculated by study authors, with confidence intervals and p-values as reported.

Ethical Considerations

This study is a synthesis of previously published, anonymised literature available in the peer-reviewed public domain; therefore, institutional review board (IRB) approval was not required. No human subjects were directly involved in this review.

Results

Study Selection and Characteristics

Our systematic search identified 12 primary studies published between 2019 and 2024 [6,7,10,12,13,16-22], encompassing 1,675 patients who sustained e-scooter-related injuries (Table 1). These studies represented substantial geographic diversity, spanning North America, Europe, the Asia-Pacific region, the Middle East and Oceania. Study designs consisted predominantly of retrospective cohort studies, with one retrospective/prospective cohort study. Amongst the 12 clinical cohort studies, 823 patients (49.1%) sustained craniofacial injuries, of whom 80 (9.7%) presented with orbital or ophthalmic injuries requiring specialised evaluation. Patient age ranged from 5 to 79 years across all studies (weighted mean = 34.5, calculated across seven studies, n = 785 [7,12,13,17-19,21]), with male patients comprising 64.3% of the overall cohort.

**Table 1:** Characteristics of Included Studies Examining Orbital and Ophthalmologic Injuries From Electric Scooter Accidents Legend: Author-derived table with data from primary studies ^a^Percentages calculated from total craniofacial injuries (Table 1) ^b^Fracture locations, multiple walls and surgical rates calculated from patients with orbital fractures ^c^Multiple walls: ≥2 orbital walls fractured concurrently ^d^Goh et al. [13] and Piccolino et al. [21] are surgical case series that enrolled only operative patients (100% surgical rate by study design) ^e^Brownson et al. [16] reported three orbital fractures, but five surgeries were performed; discrepancy suggests non-orbital procedures included ^f^Yarmohammadi et al. [18] reported individual wall fractures (52 total), but not unique patients with orbital fractures; percentages not calculable -: data not reported in study/not calculable

Study	Patients with orbital fractures (number (%))^a^	Floor fractures (number (%))^b^	Medial wall (number (%))^b^	Lateral wall (number (%))^b^	Roof (number (%))^b^	Multiple walls (number (%))^bc^	Surgical intervention (number (%))^b^
Faraji et al. (2020) [6]	42 (22.3%)	15 (35.7%)	5 (11.9%)	16 (38.1%)	6 (14.3%)	-	-
Trivedi et al. (2019) [7]	3 (5.8%)	-	-	-	-	1 (33.3%)	-
Shiffler et al. (2021) [10]	8 (21.1%)	-	-	-	-	-	-
Goh et al. (2023) [13]	9 (50%)	-	-	-	-	-	9^d^
Brownson et al. (2019) [16]	3 (9.7%)	-	-	-	-	3 (100%)	5^e^
Oksanen et al. (2020) [17]	6 (40%)	-	-	-	-	-	3
Yarmohammadi et al. (2020) [18]	-^f^	17 (-)	8 (-)	18 (-)	9 (-)	-	1
Choi et al. (2022) [19]	4 (6.2%)	3 (75%)	1 (25%)	-	-	-	-
Arbel et al. (2022) [20]	2 (0.8%)	-	-	-	-	-	-
Piccolino et al. (2024) [21]	5 (18.5%)	-	-	-	-	2 (40%)	5^d^
Suominen et al. (2022) [22]	-	-	-	-	-	-	2

Overall Orbital Fracture Prevalence

Amongst patients with craniofacial injuries, orbital fractures occurred in 12.2% (95% confidence interval (CI): 9.9%-14.9%, range: 0.8%-50.0%, n=82/672 patients across nine studies) (Table 2) [6,7,10,13,16,17,19,20,21]. This substantial heterogeneity in reported prevalence reflected important methodological differences in study populations and denominator definitions. For instance, prevalence calculations utilising a patient-based denominator yielded estimates as low as 0.8% (2/238 patients) [20], whereas those utilising a fracture-based denominator yielded estimates exceeding 24% (42/171 fractures) [6]. Studies that included all patients presenting to emergency departments with any craniofacial injury [7,10,19] reported lower orbital fracture rates (0.8%-9.7%), whereas studies focusing specifically on surgically managed maxillofacial trauma or tertiary referral populations [13] reported considerably higher rates (50%). The variation in denominators across studies, with some reporting orbital fractures as a proportion of all craniofacial injuries, others as a proportion of facial fractures only and still others restricting analysis to surgically treated cases, substantially influenced prevalence estimates and precluded direct comparability. Notably, Faraji et al. reported orbital fractures constituting 24.6% of all documented craniofacial osseous fractures (n=42/171 fractures), employing a fracture-based rather than patient-based denominator that yielded a higher apparent prevalence [6].

**Table 2:** Orbital Fracture Patterns and Surgical Management Across Included Studies Legend: Author-derived table with data from primary studies ^a^Percentages calculated from total craniofacial injuries (Table 1) ^b^Fracture locations, multiple walls and surgical rates calculated from patients with orbital fractures ^c^Multiple walls: ≥2 orbital walls fractured concurrently ^d^Goh et al. [13] and Piccolino et al. [21] are surgical case series that enrolled only operative patients (100% surgical rate by study design) ^e^Brownson et al. [16] reported three orbital fractures but five surgeries performed; discrepancy suggests non-orbital procedures included ^f^Yarmohammadi et al. [18] reported individual wall fractures (52 total), but not unique patients with orbital fractures; percentages not calculable -: data not reported in study/not calculable

Study	Patients with orbital fractures (number (%))^a^	Floor fractures (number (%))^b^	Medial wall (number (%))^b^	Lateral wall (number (%))^b^	Roof (number (%))^b^	Multiple walls (number (%))^bc^	Surgical intervention (number (%))^b^
Faraji et al. (2020) [6]	42 (22.3%)	15 (35.7%)	5 (11.9%)	16 (38.1%)	6 (14.3%)	-	-
Trivedi et al. (2019) [7]	3 (5.8%)	-	-	-	-	1 (33.3%)	-
Shiffler et al. (2021) [10]	8 (21.1%)	-	-	-	-	-	-
Goh et al. (2023) [13]	9 (50%)	-	-	-	-	-	9^d^
Brownson et al. (2019) [16]	3 (9.7%)	-	-	-	-	3 (100%)	5^e^
Oksanen et al. (2020) [17]	6 (40%)	-	-	-	-	-	3
Yarmohammadi et al. (2020) [18]	-^f^	17 (-)	8 (-)	18 (-)	9 (-)	-	1
Choi et al. (2022) [19]	4 (6.2%)	3 (75%)	1 (25%)	-	-	-	-
Arbel et al. (2022) [20]	2 (0.8%)	-	-	-	-	-	-
Piccolino et al. (2024) [21]	5 (18.5%)	-	-	-	-	2 (40%)	5^d^
Suominen et al. (2022) [22]	-	-	-	-	-	-	2

Due to limited reporting and heterogeneous denominators across studies, a formal meta-analysis was not appropriate. The following descriptive pooled estimates for fracture prevalence, anatomical distribution, multiple wall involvement and surgical intervention are described in Table 2, with the caveat that these are not meta-analytic estimates and should be interpreted cautiously given substantial methodological heterogeneity.

Anatomical Distribution of Orbital Fractures

Detailed anatomical characterisation of all four orbital walls was reported in only two studies [6,18], with one additional study [19] providing partial data (floor and medial walls only), limiting robust pooled analysis of fracture location distribution. Amongst studies reporting wall-specific fracture data with non-overlapping denominators, the orbital floor emerged as the most commonly fractured anatomical subsite, with a pooled prevalence of 39.1% (95% CI: 26.4%-53.5%, n=18/46 orbital fractures across two studies [6,19]). The lateral orbital wall demonstrated nearly equivalent frequency at 38.1% (95% CI: 25.0%-53.2%, n=16/42 in Faraji et al. [6]), followed by the orbital roof at 14.3% (95% CI: 6.7%-27.8%, n=6/42 in Faraji et al. [6]) and the medial wall with 13.0% (95% CI: 6.1%-25.7%, n=6/46 across two studies [6,19]).

The most comprehensive single-study analysis by Yarmohammadi et al. corroborated this finding, documenting orbital floor fractures in 50% of all patients with craniofacial injuries (n=17/34) [18]. Lateral orbital wall fractures were documented in 53% of patients (n=18/34) and orbital floor fractures in 50% (n=17/34), although these data could not be pooled due these high percentages reflecting the frequent occurrence of multiple concurrent orbital wall fractures within individual patients rather than mutually exclusive injury patterns. When calculated using a fracture-based denominator representing the proportion of all craniofacial fractures, Faraji et al. reported that lateral orbital wall fractures comprised 9.4% of all documented craniofacial osseous injuries (n=16/171 total fractures), orbital floor fractures in 8.8% (n=15/171), orbital roof fractures in 3.5% (n=6/171) and medial orbital wall fractures in 2.9% (n=5/171) [6]. The studies employed different denominators (total patients versus total fractures), resulting in varying reported prevalence rates.

Multiple Concurrent Orbital Wall Fractures

Multiple concurrent orbital wall fractures occurred in 54.5% of cases (95% CI: 28.0%-78.7%, n=6/11 across three studies [7,16,21]). The wide confidence interval reflected limited data availability, with only three of 12 studies [7,16,21] reporting this outcome measure. Brownson et al. [16] documented that all three patients with orbital fractures in their New Zealand cohort sustained multiple wall involvement (100%), whereas Piccolino et al. [21] reported 40% (n=2/5) and Trivedi et al. [7] reported 33.3% (n=1/3). Multiple concurrent orbital wall fractures were prevalent, reflecting high-energy anterior impact mechanisms. Specific fracture combinations were infrequently detailed in study reports, although several authors noted the common co-occurrence of orbital floor and medial wall fractures [6,16], consistent with fracture propagation through the thin lamina papyracea following initial orbital floor disruption.

Surgical Management of Orbital Fractures

Only one population-based study [17] reported surgical intervention rates with unambiguous denominators. Oksanen et al., from a maxillofacial surgery service at a university hospital, documented surgical intervention in 50% of patients with orbital fractures (n=3/6, 95% CI: 18.8%-81.2%), comprising two orbital wall reconstructions and one emergency lateral canthotomy for elevated intraorbital pressure [17]. Studies limited to surgical cohorts [13,21] reported 100% surgical rates by study design, reflecting patient selection rather than population-level surgical need. The wide variation in reported surgical rates reflects fundamental differences in study populations, specialty practice patterns and surgical indication thresholds rather than true differences in injury severity, precluding reliable estimation of surgical intervention requirements from available data.

Goh et al. reported that all nine patients with orbital involvement underwent operative intervention, with procedures including open reduction and internal fixation of zygomaticomaxillary complex fractures using two-point fixation (n=6, 66.7%) or three-point fixation (n=3, 33.3%) [13]. Surgical timing, documented by only one study, demonstrated a median interval of 10 days from injury to operation (range: 8-21 days), with 78% performed in the subacute window of 3-14 days post-injury to allow resolution of acute soft tissue oedema whilst preventing fibrotic adhesion formation [13]. Piccolino et al. reported that all five patients in their surgical cohort underwent operative reduction of displaced orbital fractures, although procedural details and timing were not specified [21]. There was substantial institutional variation in reported surgical rates, ranging from 2.9% in population-based ophthalmology cohorts (n=1/34, [18]) to 100% in surgical case series [13,18].

Vision-Threatening Complications

Vision-threatening complications were uncommon but clinically significant when they occurred, with detailed ophthalmologic outcomes systematically reported in only two of 12 studies (Table 3) [17,18]. Amongst the 57 patients with comprehensive ocular assessments, orbital compartment syndrome occurred in 3.5% (95% CI: 1.0%-11.9%, n=2/57), retinal injuries in 2.9% (95% CI: 0.5%-14.9%, n=1/34, in Yarmohammadi et al. [18]) and globe rupture in 0% (95% CI: 0.0%-6.3%, n=0/34, in Yarmohammadi et al. [18]). Traumatic optic neuropathy and hyphema were not systematically assessed or reported in any included study, precluding prevalence estimation. The remaining 10 studies [6,7,10,12,13,16,19-22], encompassing 1,618 patients, did not systematically assess or report ophthalmologic complications, representing a substantial gap in outcome documentation.

**Table 3:** Vision-Threatening Complications and Emergency Management in E-Scooter Orbital Trauma Legend: Author-derived table with data from primary studies ^a^Pooled prevalence derived from two studies providing granular ophthalmologic outcomes (Yarmohammadi et al. [18] and Oksanen et al. [17]) with a combined denominator of 57 ^b^Urgency classification: emergent (intervention required within two hours), urgent (within hours) and semi-urgent (within 24-48 hours) ^c^Posterior segment: retinal injuries (n=34) reported only by Yarmohammadi et al. [18]; Oksanen et al. did not specify and was thus excluded from this denominator [17] ^d^Yarmohammadi et al. documented reversal of NLP following timely intervention [18] CI: confidence interval, IOP: intraocular pressure, NA: not applicable, NLP: no light perception

Complication type	Polled prevalence (N/N (%))^a^	95% CI (%)	Emergency management	Urgency classification^b^
Orbital compartment syndrome	2/57 (3.5%)	1.0-11.9	Immediate lateral canthotomy/cantholysis	Emergent (90-120 minutes)
Retinal injuries (intraretinal haemorrhage)	1/34 (2.9%)^c^	0.5-14.9	Urgent vitreoretinal consult	Semi-urgent (24-48 hours)
Globe rupture	0/34 (0%)	0.0-6.3	Emergent surgical repair	Semi-urgent (24-48 hours)
Permanent vision loss	0/57 (0%)^d^	0.0-6.3	Rehabilitation/low vision aids	N/A

Orbital Compartment Syndrome

Orbital compartment syndrome, although rare (3.5%; 95% CI: 1.0%-11.9%), represented the most critical time-sensitive emergency in this review, requiring immediate lateral canthotomy and cantholysis for vision preservation. Two cases occurred across 57 systematically evaluated patients: Yarmohammadi et al. [18] at 2.9% (n=1/34) and Oksanen et al. [17] at 4.3% (n=1/23). The causative mechanism in the case in Yarmohammadi et al. was retrobulbar haemorrhage (2.9%; 95% CI: 0.5%-14.9%, n=1/34), presenting with no light perception and intraocular pressure (IOP) exceeding 50 mmHg; vision was successfully restored to baseline following emergent lateral canthotomy and cantholysis [18]. The case in Oksanen et al. similarly required emergency lateral canthotomy for elevated intraorbital pressure, although the specific aetiology was not further specified and visual outcomes were not reported [17]. Amongst the two studies with systematic ophthalmologic follow-up [17,18], no permanent vision loss was reported in patients receiving timely intervention, underscoring the critical importance of immediate recognition and surgical decompression.

Soft Tissue Injuries

Periorbital and facial soft tissue injuries were substantially more common than fractures, with prevalence varying markedly based on study population. Amongst patients sustaining any e-scooter injury presenting to emergency departments or trauma centres, facial lacerations occurred in 43.9% (95% CI: 39.9%-48.0%, n=255/581 across four studies) [6,7,16,19], facial abrasions in 41.8% (95% CI: 36.5%-47.3%, n=130/311 across two studies) [6,16] and eyelid lacerations in 12.3% (95% CI: 8.4%-17.6%, n=25/203 from one comprehensive study) [6]. However, amongst patients who had already sustained craniofacial trauma, soft tissue injury prevalence approached near-universality, with 80.1% sustaining facial lacerations, abrasions or combined soft tissue wounds (95% CI: 74.8%-84.4%, n=209/261 across two studies) [17,20]. Eyelid lacerations amongst facial trauma patients occurred in 14.7% (95% CI: 5.9%-31.4%, n=5/34), requiring specialised ophthalmologic or oculoplastic surgical consultation for assessment of canalicular involvement, levator muscle injury and full-thickness defects.

Risk Factors: Alcohol Intoxication

Alcohol intoxication was the most prevalent modifiable risk factor, documented in 33% of e-scooter injury patients (95% CI: 30.7%-35.4%, range: 7.4%-91.3%, n=523/1,585 across 12 studies) (Table 4) [6,7,10,12,13,16-22]. Substantial heterogeneity reflected study population differences and temporal patterns, with weekend nighttime studies reporting rates exceeding 85% (Oksanen et al. [17]: 91.3%, Goh et al. [13]: 85.7%) versus broader emergency department series reporting more moderate rates (Trivedi et al. [7]: 17.8%, n=16/90).

**Table 4:** Risk Factors, Temporal Patterns and Injury Circumstances Associated With E-Scooter Orbital Trauma Legend: Author-derived table with data from primary studies [6,7,10,12,13,16-22] ^a^Denominators (N) vary by row; statistics derived only from studies explicitly reporting that specific variable ^b^p-values refer to the association with severe craniofacial/orbital injury (alcohol and helmet) derived from multivariate analyses in primary studies ^c^For Shiffler et al., data regarding injury mechanisms reflect the specific craniofacial injury subgroup (N=38) rather than the total study cohort (N=165) used for demographic variables [10] ^d^Calculated from studies that differentiated "Collision With Object" from "Fall"; studies that lumped these mechanisms were excluded (Choi et al. [19] and Suominen et al. [19,22]) NA: not applicable, CI: confidence interval

Risk factor/circumstance	Contributing studies (pooled only)	Pooled total (n/N)^a^	Prevalence (%) (95% CI)	Significance^b^
Alcohol intoxication	Yarmohammadi et al. [18], Arbel et al. [20], Brownson et al. [16], Choi et al. [19], Faraji et al. [6], Goh et al. [13], Oksanen et al. [17], Piccolino et al. [21], Shiffler et al. [10], Siow et al. [12], Suominen et al. [22], Trivedi et al. [7]	523/1,585	33 (30.7-35.4)	p<0.01
Helmet use	Yarmohammadi et al. [18], Arbel et al. [20], Brownson et al. [16], Choi et al. [19], Goh et al. [13], Oksanen et al. [17], Piccolino et al. [21], Shiffler et al. [10], Siow et al. [12], Suominen et al. [22], Trivedi et al. [7]	110/1,411	7.8 (6.5-9.3)	p=0.009
Evening/night hours	Brownson et al. [16], Choi et al. [19], Oksanen et al. [17], Siow et al. [12], Suominen et al. [22], Trivedi et al. [7]	372/505	73.7 (69.7-77.3)	NA
Weekend days	Oksanen et al. [17], Suominen et al. [22], Trivedi et al. [7]	102/217	47.0 (40.5-53.6)	NA
Fall from scooter	Yarmohammadi et al. [18], Brownson et al. [16], Choi et al. [19], Shiffler et al. [10]^c^, Suominen et al. [22]	390/464	84.1 (80.5-87.1)	NA
Collision with vehicle	Yarmohammadi et al. [18], Brownson et al. [16], Choi et al. [19], Shiffler et al. [10]^c^, Suominen et al. [22]	38/464	8.2 (6.0-11.0)	NA
Collision with fixed object^d^	Yarmohammadi et al. [18], Brownson et al. [16], Shiffler et al. [10]^c^	24/255	9.4 (6.4-13.7)	NA
Other/unclear injury circumstance	Yarmohammadi et al. [18], Brownson et al. [16]	15/215	7.0 (4.3-11.2)	NA

Shiffler et al. documented the strongest association in this review: 23-fold increased odds of craniomaxillofacial injury amongst intoxicated patients (OR: 23.1, 95% CI: 7.7-69.6, p<0.01), with intoxication documented through laboratory testing or clinical assessment [10]. Arbel et al. similarly demonstrated increased odds for frontal sinus fractures (OR: 19.97, 95% CI: 1.1-361.0, p=0.04), zygomatic fractures (OR: 4.9, 95% CI: 1.21-19.79, p=0.03), open reduction and internal fixation (OR: 3.7, 95% CI: 1.24-11.03, p=0.02) and hospital admission (OR: 2.73, 95% CI: 1.21-6.16, p=0.02) [20]. Siow et al. reported that intoxicated patients had significantly higher median injury severity scores compared to sober patients (Injury Severity score (ISS): 13.0 versus 5.0, p<0.001), representing a 2.6-fold increase in injury burden [12]. The mechanism was attributed to impaired protective reflexes, with intoxicated riders unable to extend their arms to break falls, resulting in direct facial impact [10,20]. Geographic variation was notable, with Nordic studies [17,22] reporting 71%-91% intoxication rates.

Other Risk Factors and Temporal Patterns

Helmet use was critically low across all geographic regions, documented in only 7.8% of injured patients (95% CI: 6.5%-9.3%, n=110/1,411 across 11 studies) [7,10,12,13,16-22]. Geographic variation was substantial, with Israel reporting the highest helmet use at 34.1% [20] and two large United States studies reporting 0% use [7,18]. The protective effect of helmets was demonstrated by Choi et al., who reported significantly lower mean Abbreviated Injury Scale (AIS) scores for facial injuries amongst helmet wearers (p=0.009) and combined head and facial injuries (p=0.007), with the critical finding that none of five fatal cases involved helmet use [19].

Injuries demonstrated pronounced temporal clustering, with 73.7% occurring during evening and nighttime hours between 6 PM and 6 AM (95% CI: 69.7%-77.3%, range: 66.7%-82.6%, n=372/505 across five studies) [7,12,16,19,22]. Weekend clustering was particularly striking in the cohort of the study by Oksanen et al., where 82.6% of injuries occurred on Friday, Saturday or Sunday (n=19/23), with 74% specifically occurring between midnight and 6 AM [17]. The primary injury mechanism was fall from the scooter, accounting for 84.1% of injuries (95% CI: 80.5%-87.1%, n=390/464 across five studies) [10,16,18,19,22], with common contributing factors including striking curbs, drains or potholes [16], obstacles in the riding path and lack of rider experience [19]. Collision with motor vehicles accounted for 8.2% of injuries (95% CI: 6%-11%, n=38/464 across five studies) [10,16,18,19,22], although these typically resulted in more severe injury patterns. Environmental factors frequently cited included uneven road surfaces (26.6% of the cohort in the study by Siow et al. [12]), lack of dedicated bicycle lanes forcing shared roadways and poor infrastructure conditions, including tram rails and cobblestones in urban European settings [17].

Discussion

Orbital Fracture Patterns and Biomechanical Explanation

E-scooter orbital fractures show nearly equal involvement of the orbital floor (39.1%) and lateral wall (38.1%) with lower medial wall involvement (13.0%), reflecting the standing pitch-over mechanism [6,18]. During rapid deceleration, the rider's centre of mass continues forward whilst their feet remain planted on the scooter platform, rotating anteriorly around a fulcrum at their feet and resulting in direct anterior facial impact. The standing configuration with elevated centre of gravity and absent handlebar bracing predisposes riders to unprotected anterior midface trauma [6,7]. This mechanism differs fundamentally from bicycle injuries, where lateral falls predominate [11], resulting in lateral facial impacts and lateral orbital wall fractures, or arm-bracing reflexes transfer forces to upper extremities rather than the face.

Anterior impact explains why the orbital floor, the weakest structure, bears the greatest force. The lateral wall (part of the zygomaticomaxillary complex) also receives substantial force during impact [23,24]. The medial wall (lamina papyracea), despite being the thinnest orbital wall, is protected from direct anterior forces and fractures secondarily from adjacent structures [23]. Concurrent orbital floor and medial wall fractures reflect predictable fracture propagation through the lamina papyracea following initial orbital floor disruption [6,16]. This characteristic pattern should prompt systematic orbital floor assessment even with subtle external findings [25].

The relative absence of globe ruptures observed in e-scooter trauma [18] is consistent with an anterior impact mechanism, where the globe is partially protected by the supraorbital ridge and recoils posteriorly rather than experiencing the direct lateral compression typically required for rupture. However, the absence of reported globe ruptures may reflect survival bias, as patients with catastrophic injuries may not have been systematically evaluated ophthalmologically or may not have survived to presentation. The high frequency of concurrent soft tissue injuries, with periorbital lacerations and contusions approaching universality in patients with facial trauma [6], provides additional evidence of the characteristic anterior impact pattern.

Clinical Significance of Multiple Wall Fractures

More than half of orbital fractures involved multiple concurrent walls [7,16,21], carrying substantial clinical significance for surgical planning and prognostic counselling. Multiple-wall fractures indicate higher-energy impacts with greater disruption of normal orbital anatomy, increased likelihood of orbital volume changes and elevated risk of persistent functional deficits, including diplopia and enophthalmos [23,24]. Small single-wall floor fractures often resolve conservatively with spontaneous diplopia resolution and acceptable globe position [26]. However, complex multiple-wall fractures typically require operative reconstruction to restore orbital volume, release entrapped extraocular muscles and prevent globe malposition [27].

Surgical reconstruction of multiple-wall fractures presents greater technical challenges than single-wall repairs. Multiple fracture sites may require combined surgical approaches, increasing operative time and soft tissue dissection [23]. Simultaneous multiple-wall collapse distorts three-dimensional anatomy, complicating reduction and fixation. Orbital volume restoration is critical: 1-2 mL volume increases produce clinically significant enophthalmos [24]. Reconstructive material selection (autogenous bone grafts or alloplastic implants (titanium mesh and porous polyethylene)) depends on defect extent. Multiple-wall injuries often require customised implants or computer-assisted surgical planning [23].

The high prevalence of multiple-wall involvement in our review also has implications for the timing of surgical intervention. Whilst the majority of procedures in the Goh et al. [13] cohort occurred in the subacute window of 3-14 days post-injury, complex multiple-wall fractures may benefit from early intervention to prevent fibrotic contracture and adhesion formation [13]. Conversely, acute soft tissue oedema obscures anatomical landmarks, supporting delayed repair once oedema resolves [24].

Orbital Compartment Syndrome: Time-Critical Emergency

Orbital compartment syndrome, documented in 3.5% of systemically evaluated e-scooter patients [17,18], represents the only time-critical emergency requiring immediate intervention to prevent permanent vision loss. Intraorbital pressure rises, typically from retrobulbar haemorrhage or haematoma formation, compresses the optic nerve and central retinal artery, causing progressive optic nerve ischaemia that becomes irreversible after 90-120 minutes without decompression [28,29]. Yarmohammadi et al. documented a patient with no light perception and IOP > 50 mmHg who achieved complete visual recovery following emergent lateral canthotomy and cantholysis, demonstrating reversibility with prompt treatment [18].

Emergency physicians and ophthalmologists must recognise cardinal signs: severe orbital pain, proptosis, tense eyelids, decreased or absent vision, elevated intraocular pressure (typically >40 mmHg) and relative afferent pupillary defect [28,29]. Any e-scooter patient with periorbital trauma and vision complaints requires immediate intraocular pressure measurement; this assessment should not be delayed for specialist consultation. Suspected orbital compartment syndrome requires immediate bedside lateral canthotomy and cantholysis without delay for imaging or subspecialty consultation, a mandate that applies universally, including in resource-limited settings where transfer delays would otherwise result in permanent blindness [30].

Lateral canthotomy can be performed by trained emergency physicians and ophthalmologists. Bedside performance eliminates operating room delays and prioritises vision salvage over cosmetic concerns, which can be addressed through delayed reconstruction [30]. These outcomes support emergency physician training in lateral canthotomy. Emergency physicians require hands-on training in canthotomy technique with procedural equipment readily available [30]. All on-call ophthalmology physicians must be capable of independent performance given acute time constraints. Permanent blindness from a potentially reversible condition represents an unacceptable outcome when effective intervention exists.

Broader Vision-Threatening Complications

Retrobulbar haemorrhage (occurring in 3.5% of patients) requires serial IOP monitoring for 24-48 hours, as delayed expansion or rebleeding can occur hours after initial injury [18,28]. Even patients with initially normal IOP can develop compartment syndrome if haemorrhage accumulates [28]. Retinal injuries, including intraretinal haemorrhages (2.9% of patients), indicate posterior pole force transmission and warrant dilated fundoscopy to exclude retinal tears, macular holes or commotio retinae that may not be visible on computed tomography (CT) imaging [18]. Lack of systematic ophthalmologic assessment in 85% of studies suggests true prevalence exceeds documented rates.

Long-term functional outcomes are inadequately assessed across most studies. Persistent diplopia, chronic enophthalmos requiring revision surgery, long-term visual acuity changes and vision-related quality of life are rarely assessed beyond immediate postoperative follow-up. Retinal injuries may present with delayed complications, including retinal detachment, weeks to months after trauma, requiring extended surveillance not systematically implemented [18].

Surgical Management Variation

Surgical rates vary substantially by study design, 5.9% in population-based ophthalmology cohorts [18], 20% in maxillofacial surgery [17] and nearly 100% in surgical case series [21], reflecting differences in study design, institutional thresholds and specialty-specific practice rather than true injury severity. This heterogeneity reflects absent standardised surgical indication criteria for e-scooter orbital trauma. The 3.4-fold difference between surgical specialties reflects divergent philosophies: Burnstine [26] noted that management of orbital fractures varies considerably across specialties, with conservative management often appropriate for isolated fractures without functional deficit, whilst Joseph and Glavas [23] observed that surgical indications may differ based on specialty focus, with ophthalmology prioritising visual function and maxillofacial surgery incorporating both functional and aesthetic reconstruction considerations.

Traditional indications for orbital fracture repair include significant enophthalmos (>2 mm), persistent diplopia beyond two weeks, large floor defects (>50% or >2 cm²), positive forced duction testing and acute enophthalmos indicating large orbital volume expansion [26,27]. Application requires clinical judgment, varying by training background, resources and patient preferences. The Burnstine criteria [26], developed for general orbital fractures, may not capture e-scooter-specific patterns, where multiple-wall involvement may warrant earlier intervention.

Resource availability substantially influences surgical decision-making, with tertiary referral centres having immediate access to specialised equipment, intraoperative imaging and customised implants that facilitate complex reconstructions [23], whilst community hospitals may need to transfer complex cases or rely on more conservative management. Most procedures occur 7-14 days post-injury [13,17], balancing allowing soft tissue oedema resolution against preventing fibrotic changes, although optimal timing remains uncertain and varies by fracture complexity and patient-specific factors [24].

The lack of standardised protocols represents both a limitation and an opportunity for quality improvement. Future research should focus on developing evidence-based guidelines incorporating injury mechanisms, fracture characteristics and patient factors. Prospective comparative studies of operative versus conservative management for specific fracture patterns would address current evidence gaps. Until such data become available, surgical decisions should be individualised with comprehensive assessment, informed consent and multidisciplinary input when available.

Alcohol as the Primary Modifiable Risk Factor

Alcohol intoxication, present in 33% of e-scooter injury patients overall (from our pooled analysis) and exceeding 85% in weekend nighttime cohorts [13,17], represents the most important modifiable risk factor. This substantially exceeds that observed in motor vehicle trauma patients (approximately 30% in trauma registries) [31] and injured bicyclists (15%) [32], establishing intoxicated e-scooter use as particularly dangerous. Alcohol intoxication is associated with 23-fold increased odds of craniomaxillofacial injury [10], comparable to unrestrained motor vehicle occupants or motorcycle crashes. This association reflects biomechanical reality: alcohol impairs neuromuscular coordination necessary for protective responses during falls [10].

Alcohol increases the risk of orbital injury in e-scooter accidents through multiple mechanisms. Impaired judgment leads to riskier riding behaviours, including excessive speed, navigation of challenging terrain and operation in poor visibility conditions that sober riders would avoid [10]. Intoxicated riders show measurably slower reaction times and reduced arm extension, failing to transfer impact to extremities [10]. Inability to execute arm extension results in direct anterior facial impact concentrating forces on the orbit. Absence of distal extremity injuries in severe facial trauma [12] supports this mechanism.

These findings demand policy responses comparable to drunk driving prevention. Most jurisdictions apply blood alcohol limits (typically 0.08%) to e-scooters, yet enforcement is minimal due to rental models, the absence of registration requirements and limited police resources for traffic enforcement. E-scooter rental systems lack effective mechanisms to prevent intoxicated operation, unlike automobiles with checkpoints and license suspensions. Possible interventions include breathalyser-equipped unlocks, time-of-day rental restrictions or geofencing in entertainment districts. Implementation challenges include user circumvention (having sober friends unlock devices), technical reliability with breathalyser integration and discrimination concerns with geographic restrictions.

Educational campaigns targeting young adults (the primary e-scooter demographic) should emphasise that intoxicated riding carries comparable risks to drunk driving, despite lower speeds and perceived safety of remaining on pavements and in bicycle lanes. Bars and restaurants in high e-scooter usage areas can serve as prevention partners, posting intoxication warnings and offering alternative transportation incentives. Emergency department discharge instructions for patients treated for e-scooter injuries should include explicit warnings about alcohol use and riding, potentially leveraging the "teachable moment" of injury to modify future behaviour. Addressing alcohol-impaired e-scooter use requires a multipronged approach: legislative action, enhanced enforcement, public education and social norm changes, making intoxicated riding culturally unacceptable.

Helmet Use and Prevention Strategies

The review reported a helmet use rate of 7.8% overall and 0% in US cohorts [7,18]. Standard helmets provide limited midface and orbital protection but offer benefits warranting promotion. Helmets substantially reduce concurrent traumatic brain injury, which occurred alongside orbital trauma in a significant proportion of cases in our review [22]. Skull base and cranial vault fractures occurred exclusively in non-helmeted riders [20], providing evidence for helmet protection against severe outcomes. None of five fatal cases involved helmet use [19]; whilst not reaching statistical significance due to small numbers, it suggests helmets may prevent deaths despite limited facial protection.

Helmet mechanisms extend beyond direct impact absorption. Helmeted riders may approach riding more cautiously, correlating helmet use with overall safer behaviour, including lower speeds and safer routes. Helmets may reduce impact velocity by providing larger, more compliant surfaces that increase deceleration time and distance. Some modern helmet designs incorporate extended coverage or integrated face shields that provide substantially more facial protection than traditional bicycle helmets, although adoption of these designs remains limited due to cost, convenience and aesthetic concerns. Full-face helmets provide complete orbital protection; however, adoption barriers include expense (often exceeding $200), storage incompatibility with rental models and social acceptability concerns.

Multiple barriers impede helmet adoption in e-scooter use. The rental business model, where users access scooters spontaneously without advance planning, conflicts with personal helmet ownership and carrying. Rental companies may face challenges, including theft, hygiene concerns over shared helmets and low uptake. Relatively low speeds (15-20 mph) create a false sense of safety, leading users to underestimate injury risk. Mandatory helmet laws face opposition from industry and civil liberties advocates, although several cities have implemented requirements.

Complementary prevention strategies merit consideration. Speed limiters reducing maximum velocity to 12-15 mph in high-pedestrian areas could decrease impact forces. Geofencing technology that automatically reduces speed or disables scooters in high-risk zones (steep hills, busy intersections and entertainment districts) is feasible and is implemented in some jurisdictions. Enhanced rider education, potentially including mandatory online safety tutorials before first rental activation, could improve awareness of injury risks and proper riding technique. Infrastructure improvements, including dedicated e-scooter lanes separated from both vehicular traffic and pedestrians, improved street surface maintenance to eliminate potholes and curb hazards and enhanced nighttime lighting in high-usage corridors, would address environmental factors identified in our review [12,16]. Effective prevention requires coordinated efforts across rental companies, municipal governments, traffic enforcement and emergency medicine providers.

Temporal Clustering and Public Health Implications

Temporal clustering of 73.7% evening/nighttime injuries (pooled analysis results) and 82.6% weekend injuries [17] enables prevention targeting and healthcare resource planning. This pattern directly mirrors alcohol consumption patterns in urban entertainment districts, with peak injury occurrence between midnight and 6 AM coinciding with bar closing times [17,22]. Injuries concentrate in predictable high-risk windows when intoxicated riders navigate home. The clustering enables targeted enforcement during weekend evening hours to identify and intervene with impaired riders before crashes occur [17]. Some jurisdictions have implemented "safe ride" programmes providing subsidised alternative transportation to reduce impaired e-scooter use.

Temporal patterns require adjusted emergency department and ophthalmology staffing. Emergency departments should anticipate peak presentations of e-scooter facial trauma during late-night and early-morning hours on weekends, requiring adequate coverage by providers comfortable managing orbital emergencies [28,30]. On-call ophthalmology services must be prepared for urgent consultations during these high-risk periods, with clear protocols for timely response to vision-threatening complications. Overnight imaging availability may be limited, potentially delaying diagnosis. Urban hospitals with high e-scooter volume should adjust resource allocation and staffing models accordingly.

Rental companies can implement policy modifications during high-risk periods. Time-of-day pricing that increases costs during late-evening hours might deter intoxicated riding whilst maintaining availability during safer daytime periods. Automatic speed reduction after 10 PM in entertainment districts could limit injury severity even if it cannot prevent all crashes. Push notifications to active users during high-risk hours warning about increased accident risk and encouraging alternative transportation represent feasible technological interventions. Some cities have implemented mandatory parking corrals in entertainment districts to prevent scooters from cluttering sidewalks whilst simultaneously concentrating them in areas where intoxicated riders are more likely to make poor decisions, creating potential for targeted prevention messaging. Policy interventions require evaluation comparing pre- and post-implementation injury rates to identify effective strategies.

Comparison With Existing Literature

Our findings align with the broader e-scooter injury literature [1,4] whilst providing novel orbital-specific insights. Our overall craniofacial injury prevalence (approximately 49%) is consistent with prior systematic reviews [1,33], and the male predominance (64%) and young adult age distribution match established patterns. The strong association between alcohol intoxication and injury severity was noted in general e-scooter studies [5,22]; our review provides the first quantification specific to orbital trauma, with a 23-fold odds ratio [10]. Critically low helmet use confirms prior reports [1,4], although our synthesis benefits from larger sample sizes across more diverse geographic settings.

Several findings represent novel contributions. The predominance of orbital floor and lateral wall fractures over medial wall involvement, whilst biomechanically expected [23], had not been quantitatively demonstrated in e-scooter-specific literature. The high prevalence of multiple concurrent wall fractures (54.5%) exceeds that in general facial trauma populations [24] and distinguishes e-scooter accidents as particularly high-energy mechanisms compared to other orbital fracture causes. The documentation of orbital compartment syndrome in 3.5% of systematically evaluated patients [17,18], with successful visual recovery following emergency intervention, provides the first evidence that this complication occurs at clinically meaningful rates in e-scooter trauma.

Our review identifies critical literature gaps: long-term functional outcomes, including persistent diplopia and vision-related quality of life, remain virtually unreported; detailed ophthalmologic assessment is absent in 85% of studies despite being essential for functional evaluation; and economic analyses quantifying societal costs (direct medical expenses, lost productivity and disability) are entirely absent despite their importance for policy and prevention resource allocation. These gaps represent priority areas for future investigation.

Limitations

This review has important limitations warranting careful interpretation. All studies except Piccolino et al. [21] used a retrospective observational design, introducing inherent biases characteristic of this methodology. Retrospective data collection relies on variable documentation quality across institutions and providers. Incomplete documentation of key variables, including precise visual acuity measurements, specific fracture configurations and alcohol assessment methods, limits analysis depth. Selection bias is inevitable as only emergency or trauma centre patients are captured; those with minor injuries or no care-seeking are unrepresented. This severity bias likely inflates the apparent frequency of complex fractures and surgical interventions relative to the true denominator of all e-scooter riders sustaining any degree of orbital trauma.

Heterogeneity across multiple dimensions prevented meta-analysis and complicates the interpretation of pooled estimates. Definitions of what constitutes an "orbital injury" varied from studies including any periorbital soft tissue trauma to those restricting analysis to documented fractures visible on CT [25]. Denominator choices differed markedly, with some studies reporting orbital fractures as a proportion of all e-scooter injuries [7,12], others as a proportion of craniofacial injuries only [6,18] and still others focusing exclusively on surgically treated cases [13,21]. Imaging protocols varied, with some institutions performing dedicated orbital CT with thin-slice reconstructions on all facial trauma patients, whilst others used clinical examination alone, potentially missing non-displaced fractures. Surgical thresholds and indications varied substantially across institutions and specialties, with ophthalmology, oculoplastic surgery and maxillofacial surgery services applying different criteria [26,27] that preclude meaningful comparison of surgical rates.

The included studies predominantly originated from high-income countries in North America, Europe and the Asia-Pacific region, with minimal representation from Latin America, Africa or other developing regions where e-scooter adoption is rapidly increasing. Healthcare system differences, traffic patterns, road infrastructure quality and cultural factors affecting alcohol consumption and helmet use vary across regions, limiting applicability to underrepresented areas. Most data originated from urban academic medical centres or level I trauma centres [6,7,12], introducing referral bias not reflecting injury patterns seen in community or rural settings.

Data gaps in long-term outcomes limit clinical utility. Visual acuity at presentation and follow-up visual assessment were incompletely reported. Persistent diplopia, the most functionally significant long-term complication [26], was not systematically assessed in any study beyond the immediate postoperative period. Rates of revision surgery for residual enophthalmos, malposition or persistent extraocular motility deficits remain unknown. Patient-reported outcome measures (satisfaction, with facial appearance, vision-related quality of life and functional disability) were absent from all included studies. Cost data (medical expenses, lost work productivity and disability-related costs) were reported in only one study.

Publication bias favours larger case series from high-volume centres over smaller series or negative findings. Studies reporting high injury rates or severe complications may be preferentially published due to perceived novelty or clinical importance, whilst studies finding low complication rates may remain unpublished. Language bias is inherent in our restriction to English-language publications, potentially excluding relevant data from non-English literature, particularly from Europe and Asia, where e-scooter adoption has been substantial. The absence of a prospectively registered protocol and our decision to perform narrative synthesis rather than quantitative meta-analysis, whilst justified by the substantial heterogeneity in study designs and outcome measures [34], nonetheless limits the rigor and reproducibility of our findings compared to a formal systematic review with pre-specified analytical plans.

Future Research Directions

Addressing the limitations identified in this review requires a coordinated research agenda spanning multiple methodological approaches. Prospective multicentre cohort studies with standardised assessment protocols are the priority: comprehensive ophthalmologic evaluation at multiple time points including initial presentation, hospital discharge, one month, three months, six months and 12 months later, using validated instruments including best-corrected visual acuity, Hess screen testing for diplopia quantification, Hertel exophthalmometry for globe position measurement and standardised facial photography with three-dimensional photogrammetry when available. Vision-related quality of life should be assessed with NEI-VFQ-25 [35] and diplopia-specific questionnaires. Facial appearance satisfaction should be measured using FACE-Q [36], which provides a psychometrically validated assessment of appearance-related quality of life following facial trauma. Long-term outcomes studies extending beyond one year are essential to capture late complications, including delayed enophthalmos, progressive diplopia and revision surgery requirements, with stratification by fracture pattern, surgical intervention and patient factors to identify prognostic indicators informing clinical decision-making. Comparative effectiveness research evaluating surgical approaches through randomised controlled trials or propensity-matched observational studies should incorporate health economic analyses quantifying costs and cost-effectiveness.

Comprehensive economic analyses are essential for policy decision-making, quantifying direct medical costs and indirect costs, including lost productivity and caregiver burden, using standard health economics methodologies. Cost-effectiveness analyses comparing prevention interventions would identify strategies offering optimal value and justify prevention spending. Biomechanical research incorporating finite element analysis, crash reconstruction studies and cadaveric impact testing could elucidate injury mechanisms and inform protective equipment design through collaboration between trauma surgeons, engineers and manufacturers. Prevention research evaluating real-world interventions using natural experiment designs and randomised trials is essential, requiring collaboration across academic institutions, industry partners and policymakers to assess mandatory helmet laws, technological interventions and educational programmes targeting behaviour modification.

Clinical Practice Implications

These findings have several immediate implications for emergency medicine, ophthalmology and oculoplastic surgery. Emergency departments should implement standardised assessment protocols for all e-scooter facial trauma presentations, including systematic measurement of visual acuity in both eyes, assessment of extraocular motility in all cardinal gazes with specific attention to diplopia, intraocular pressure measurement using tonometry, pupillary examination for relative afferent pupillary defects and external examination for periorbital ecchymosis, enophthalmos and globe malposition [28,29]. Any patient presenting with vision changes, elevated intraocular pressure exceeding 30 mmHg, severe proptosis or clinical concern for orbital compartment syndrome requires immediate ophthalmology consultation without delays for imaging completion. Emergency physicians should be trained to perform emergency lateral canthotomy when ophthalmology is not immediately available and compartment syndrome is suspected, as delays affect visual prognosis [30].

Imaging decision-making should follow a risk-stratified approach. Patients with any periorbital findings on examination, mechanism concerning for high-energy impact or subjective visual complaints warrant dedicated orbital CT with thin-slice coronal and sagittal reconstructions to identify non-displaced fractures that might be missed on standard facial CT protocols [25]. Ophthalmology consultation should be obtained for all patients with orbital fractures, regardless of visual symptoms, to establish baseline assessment and ensure appropriate follow-up for delayed complications.

Patient counselling should address domains such as recovery trajectory, warning signs warranting urgent re-evaluation, activity restrictions during healing and complication risks. Patients should be explicitly warned about persistent diplopia risk, particularly with complex fractures [26], and counselled that visual symptoms may not fully resolve despite anatomically successful fracture healing. Discharge instructions must include return precautions for worsening vision, severe pain or increasing proptosis indicating delayed complications. All patients should receive standardised written education regarding alcohol-impaired riding risks [10] and helmet use importance [19], potentially leveraging the "teachable moment" of injury to modify future behaviour. These clinical practice recommendations, informed by the comprehensive evidence synthesis in this review, aim to optimise outcomes for this emerging injury pattern affecting thousands of patients annually in urban centres worldwide.

## Conclusions

This systematic synthesis establishes orbital trauma as a common complication of electric scooter accidents, characterised by distinct biomechanical patterns reflecting the standing-posture "pitch-over" mechanism. Direct anterior midface impacts explain the predominance of orbital floor and lateral wall fractures, with multiple concurrent wall involvement indicating high-energy trauma. While orbital compartment syndrome remains infrequent, it represents a time-critical emergency requiring immediate recognition and intervention through systematic intraocular pressure assessment and bedside canthotomy when indicated. Current surgical management varies substantially across specialties and institutions, highlighting the critical need for standardised treatment protocols. These variations reflect divergent approaches to functional versus aesthetic reconstruction and differing thresholds for surgical intervention.

Alcohol intoxication emerges as the predominant modifiable risk factor, with pronounced temporal clustering during evening and weekend hours providing clear prevention targets alongside critically low helmet utilization rates. However, critical evidence gaps persist regarding long-term functional outcomes, optimal surgical indications and prevention strategy effectiveness. Future research must employ prospective designs with standardised outcome assessments and extended follow-up to address these deficiencies. Mitigating the growing public health burden of e-scooter orbital trauma requires coordinated multipronged strategies encompassing technological solutions, enhanced enforcement and infrastructure modifications. Collaborative efforts across policymakers, rental companies, healthcare systems and urban planners are urgently needed to implement these interventions effectively.
